# A comparison of hallux valgus angles assessed with computerised plantar pressure measurements, clinical examination and radiography in patients with diabetes

**DOI:** 10.1186/1757-1146-7-33

**Published:** 2014-07-21

**Authors:** Daniël MC Janssen, Antal P Sanders, Nick A Guldemond, Joris Hermus, Geert HIM Walenkamp, Lodewijk W van Rhijn

**Affiliations:** 1 Orthopaedic Surgery Department, Maastricht University Medical Centre, P. Debyelaan 25, Maastricht, The Netherlands; 2 Dorati Consultancy for Feet and Health, Los Angelesstraat 74, The Hague, The Netherlands; 3 University of Technology Delft, Faculty Electrical Engineering, Mathematics & Computer Science, Department Man-machine Interaction, University of Technology Delft, Mekelweg 4, Delft, The Netherlands

## Abstract

**Background:**

Hallux valgus deformity is a common musculoskeletal foot disorder with a prevalence of 3.5% in adolescents to 35.7% in adults aged over 65 years. Radiographic measurements of hallux valgus angles (HVA) are considered to be the most reproducible and accurate assessment of HVA. However, in European countries, many podiatrists do not have direct access to radiographic facilities. Therefore, alternative measurements are desired. Such measurements are computerised plantar pressure measurement and clinical goniometry. The purpose of this study was to establish the agreement of these techniques and radiographic assessments.

**Methods:**

HVA was determined in one hundred and eighty six participants suffering from diabetes. Radiographic measurements of HVA were performed with standardised static weight bearing dorsoplantar foot radiographs.

The clinical goniometry for HVA was measured with a universal goniometer. Computerised plantar pressure measurement for HVA was executed with the EMED SF-4® pressure platform and Novel-Ortho-Geometry software. The intra-class correlation coefficients (ICC) and levels of agreement were analysed using Bland & Altman plots.

**Results:**

Comparison of radiographic measurements to clinical goniometry for HVA showed an intraclass correlation coefficient (ICC) of 0.81 (95% confidence interval, 0.76 to 0.86; *p<0.001*). Radiographic measurement versus computerised plantar pressure measurement showed an ICC of 0.59 (95% confidence interval, 0.49 to 0.68; *p<0.001*). In addition, clinical goniometry versus computerised plantar pressure measurement showed an ICC of 0.77 (95% confidence interval, 0.70 to 0.82; *p<0.001*). The systematic difference of the computerised plantar pressure measurement compared with radiographic measurement and clinical goniometry was 7.0 degrees (SD 6.8) and 5.2 degrees (SD 5.0), respectively. The systemic difference of radiographic measurements compared with clinical goniometry was 1.8 degrees (SD 5.0).

**Conclusions:**

The agreement of computerised plantar pressure measurement and clinical goniometry for HVA compared to radiographic measurement of HVA is unsatisfactory. Radiographic measurements of HVA and clinical goniometry for HVA yield better agreement compared to radiographic measurements and computerised plantar pressure measurement. The traditional radiographic measurement techniques are strongly recommended for the assessment of HVA.

## Background

Healthcare professionals involved in the treatment of foot and ankle disorders are confronted with a high prevalence of hallux valgus deformity. The prevalence of this deformity increases from 3.5% in a healthy population of adolescents to 35.7% in adults aged over 65 years [1-5].

The most frequent complaint of patients with hallux valgus is pain as a result of pressure between the bunion and the shoe or between the first and second toe. Pain can also be caused by overloading the plantar aspect of one or more metatarsophalangeal joints [4,6]. Hallux valgus and bunion are separate entities, in contrast to the lay use of the term bunion to equate hallux valgus. We describe a bunion as the prominence at the medial side of the ball of the foot, which is formed by the protruding metatarsal head and in many cases by additional bone formation, swollen skin and sometimes a bursa. Elevated pressure on the skin as a result of inter-digital contact and shoe wear could also lead to ulceration. This is a serious complication in patients with insensate feet, for example due to diabetic neuropathy. These patients need specialised attention and adequate intervention [1,3]. Some authors use the term ‘clinical hallux valgus’ when the deformation has developed to a certain severity that causes complaints [7-9].

A rough estimation of the hallux deviation is normally determined through physical inspection of the foot. This estimation is used as a measure for clinical classification and decision making. Accordingly, hallux deviation is used to estimate the severity of the hallux valgus, to evaluate progression in time and to evaluate the effect of an intervention. Together with the degree of pain, as the most important indication criterion for treatment decisions, the hallux deviation is also part of the selection criteria for conservative or operative treatment. In 1984, the Research Committee of American Orthopaedic Foot and Ankle Society developed a guideline for the study of hallux valgus by physical examination and radiographic measurement [10]. The most commonly used indicator for hallux valgus deformity is the hallux valgus angle (HVA, synonym: hallux angle) assessed by means of radiography [11].

Until now, it is not known what the best method is for the assessment of the hallux valgus angle in clinical settings. Various methods to measure the HVA such as radiographic measures and clinical goniometry are used in clinical practice. The measurement of the bone alignment through radiographs, is considered to be the most reproducible and accurate assessment of hallux valgus. Therefore, this could be appreciated as a ‘gold standard’ [12,13]. On radiographs, the hallux angle is measured and defined as the angle between the longitudinal axes of the proximal phalanx of the hallux and the first metatarsal [7]. For the assessment of hallux valgus through radiographs, the inter-observer intraclass correlation as well as the intra-observer intraclass correlation will increase with the use of exact guidelines [12,13].

Since the availability of more modern techniques, like computerised plantar pressure measurement for HVA, it would be of great value to know whether alternative measurements are equally reproducible. These alternatives have the additional benefit that repeated irradiation during follow-up could be avoided. Professionals without direct access to radiographic facilities, like most podiatrists in European countries, could also use these measurements. Clinical goniometry is another method to assess the degree of the valgus deviation. Deviation of the hallux established with measurements on contours of pressure profiles of footprints can be obtained with a Harris and Beath (ink) foot printing mat, a Podotrack (a footprint mat with a carbon-paper sheet for pressure gradient measurement), or with electronic systems for computerised plantar pressure measurements [14-16].

In an earlier study, Sanders et al. [8] found a statistically significant correlation between measurements of HVA with weight-bearing radiographs and static ink footprints in 11 patients (r_s_=0.9, *p*=0.004). These authors suggested that a HVA of 8 degrees, measured through footprints, could substitute a radiographic HVA of 15 degrees, and could function as a cut-off value for asymptomatic hallux valgus and clinical hallux valgus. Existing literature only describe the correlation of radiographs and footprints or pressure measurement for foot parameters other than HVA [17,18].

A correlation of HVA measured by radiographs and measured by photographic measurement or a grading scale is described by Nix et al. [19]. They investigated the reliability and concurrent validity of photographic measurements of the hallux valgus angle compared to radiographs and found an intraclass correlation coefficient greater than 0.96. Menz et al. [20] investigated the correlation of a clinical assessment grading scale (the Manchester scale scores) with hallux valgus measurements obtained from radiographs. They found a high correlation (Spearman’s rho = 0.73, *p*<0.01). Garrow et al. [21] found a kappa score of 0.86 for the interobserver repeatability for the Manchester scale. Another grading scale comparative to the Manchester scale was investigated by Roddy et al. [22]. They found a kappa score of 0.82 for the observer repeatability for a five-grade hallux valgus scale developed from a photograph of a normal foot.

In order to establish alternative measurement of the HVA without radiographs, this study aims to assess the level of agreement of the hallux valgus deviation measured through radiographs compared to clinical goniometry and computerised plantar pressure measurement.

## Methods

To compare three measurement methods for HVA, patients from the outpatient clinic of the Maastricht University Medical Centre, suffering from diabetes and with or without hallux valgus deviation, were included. These patients were randomly selected as part of a larger project concerning diabetic foot problems by using the opaque envelope method. Neither our clinical experience, nor literature search led to arguments that the presence of diabetes has significantly influenced the study methods or results. The included participants did not have any specific foot deformities irrespective of hallux valgus deformity. There were no signs of Charcot neuroarthropathy or significant pes planus valgus. The inclusion criteria were: diabetes mellitus type 1 (insulin dependent) or type 2 (non-insulin dependent), age between 30 and 75 years and ability to perform daily-life activities without supporting devices. The exclusion criteria were: a history of rheumatoid arthritis, severe foot trauma, foot ulceration, surgery of the foot and/or a foot deformity other than spread foot, hallux valgus or lesser toe deformities.

Before the start of the study, participants were informed about all study procedures and possible risks. The Research Ethical Committee of the Maastricht University Medical Centre approved the study. Only one trained examiner (NAG) performed the physical examination on all the participants’ feet. Each participant was tested on one day. The examiner is an experienced clinical researcher of diabetic foot complications: i.e. five years outpatient clinic experience. Data were obtained through radiographic measurements of HVA, clinical goniometry for HVA (mean of three measurements) and computerised plantar pressure measurement for HVA. The data of the radiographic measurements and the computerised plantar pressure measurements were evaluated after the last participant was measured, to prevent examiner’s bias during the collection of data.

### Radiographic measurement

Measurements from standardised static weight-bearing foot radiographs have shown to be an objective and reliable way of assessing both bony structure and soft tissue dimensions [21,23-27]. In the literature, radiographic measurement of HVA is described as the “gold standard” and commonly used by foot and ankle specialists [12,13]. An additional advantage of radiography is the possibility to evaluate more important information of the bony structures of the foot, especially preoperative. For example the quality of the joint surfaces and also other angles like the intermetatarsal angle, the distal metatarsal articular angle and the proximal phalangeal articular angle are relevant [10]. Weight-bearing dorsoplantar radiographs were taken with the participant in normal standing position on a platform with the central beam aimed with 20 degree anterior tilt to the vertical directed at the navicular (55 kV, 12 mAs) from a distance of 150 cm. This radiographic protocol was previously described by Cavanagh et al. [23]. A radio-opaque marker (L-shaped 8×12×20 mm) was placed on the radiographic plate in order to judge afterwards whether scale correction was necessary. All radiographs were taken by the same radiographer, using the same equipment and settings.

**Figure 1:**
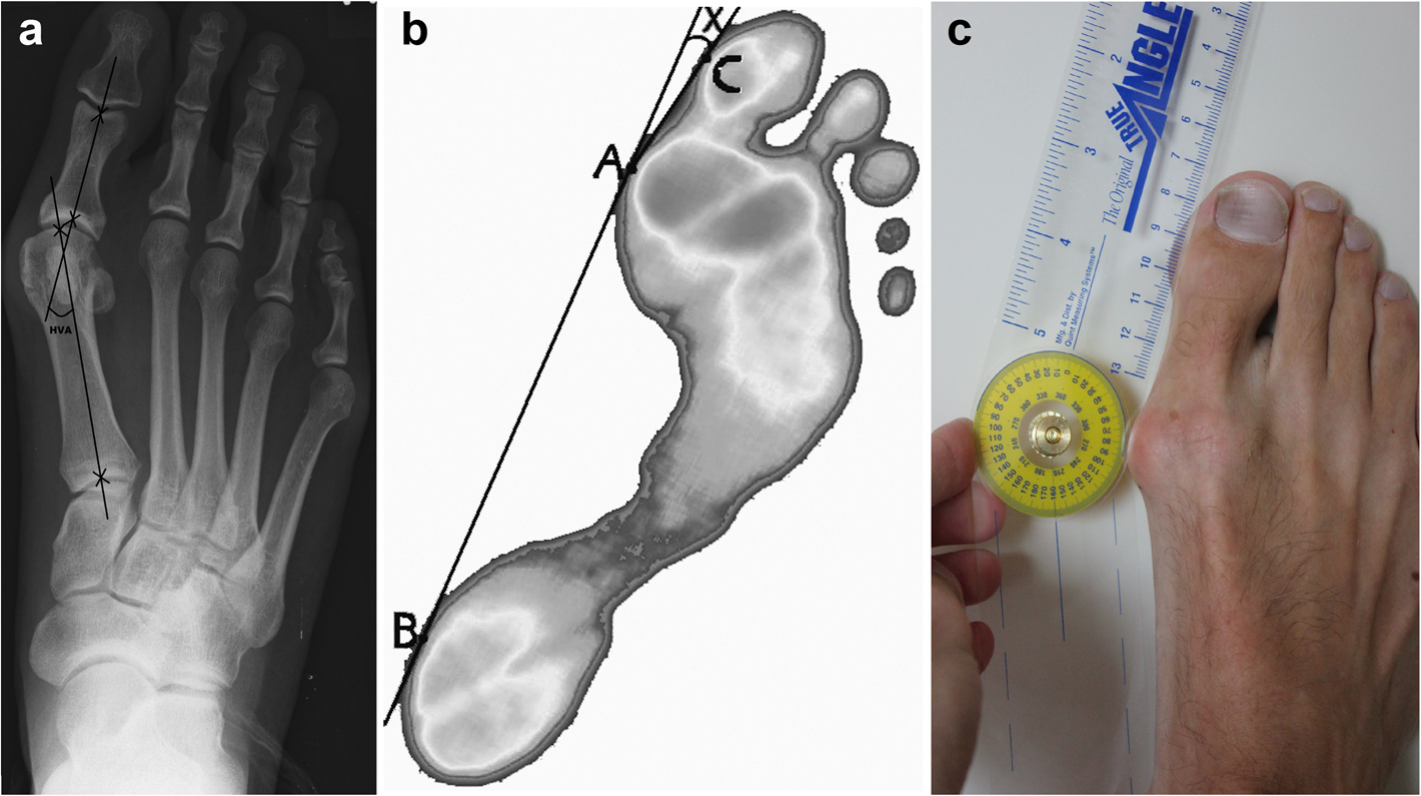
HVA measurement through radiography (a) according to the method of Mitchell et al., through computerised plantar pressure (b) and through clinical examination (c).

To measure the HVA, the angle between the longitudinal axes of the first metatarsal and the proximal phalanx of the hallux was assessed. To determine the longitudinal axis of the first metatarsal the method as described by Mitchell et al. [28] which was further evaluated by Schneider et al. [13,29] was used. According to this method, a line is drawn connecting the centre of the articular surface of the first metatarsal head and the centre of the proximal articulation. A second line connecting the centre of the proximal articular surface of the first proximal phalanx and the centre of the distal end of the diaphysis of the proximal phalanx is drawn as the longitudinal axis of the first proximal phalanx (Figure 1a) [29]. According to the literature, this method is highly reliable. In addition, this is accepted for comparative studies in pre- and postoperative conditions [12,13,29]. The HVA was manually established with a goniometer measuring the angle between these two longitudinal axes. The HVA was determined once.

### Computerised plantar pressure measurement

An EMED SF-4 pressure sensitive platform (Novel, Munich) was used to quantify the barefoot plantar pressures of the participants’ feet. This 420×417 mm dimension pressure plate, integrated into a walkway of six meter length, consists of an active sensor area of 360 mm×190 mm and a matrix of 2736 high quality capacitance sensors. Each sensor has a surface area of 0.25 cm^2^ and can record pressure from 0 to 127 N/cm^2^ during posture or locomotion. The data were collected at 50 samples per second and analysed on a microcomputer [30,31].

In this study we performed the pressure measurements according to a dynamic first-step method [333-35]. Barefoot participants were positioned at the start of the walkway, and were instructed to line up their left foot with the start line. They began the trial with a right step on the platform and continued their trial with a left step behind the platform. Barefoot peak pressure was estimated by calculating the mean over the readings of five trials [30,32-34]. Based on this peak pressure, the program measured the HVA of the foot. Because of independence of measurements only the right foot was chosen for statistical analysis.

Using the Novel-Ortho software, peak pressure was quantified for different regions of the foot. In addition, a custom-developed Windows-based program (Novel-Ortho-Geometry) was used to calculate foot angles including HVA [31,35,36]. The Novel-Ortho-Geometry software calculates geometric parameters of the foot from the pressure distribution measurement. All calculations are based on the peak pressure values, which give a representation of the sensors touched or activated during the complete roll-over of the foot. The HVA was assessed using the Novel-Ortho-Geometry program. A line is drawn tangent to the medial aspect of the pressure print contour of the ball of the foot (A) and the heel (B) (Figure 1b). Another tangent is drawn to A and the medial aspect of the pressure print contour of the pulp of the hallux (C). The software measured HVA as the angle (X) between the two lines AB and AC [31,36].

### Clinical goniometry

A 360 degree, clear plastic universal goniometer with a locking device (Quint 7″ True Angle, Quint Measuring systems, San Ramon, USA) was used for measurements of the HVA [27,37,38]. This angle was measured using the goniometer of the barefoot participants whose feet were weight-bearing in a normal standing position. The centre of rotation of the goniometer was placed on the MTP joint-space on the medial contour of the foot. One arm of the goniometer was placed parallel to the medial contour of the first metatarsal and the other parallel to the medial contour of the proximal phalanx of the hallux: according to the guidelines of the American Academy of Orthopaedic Surgeons [27] (Figure 1c). The goniometer scale was accurate to one degree. A mean was calculated out of three measurements.

### Statistical analyses

Data were analysed by three investigators, including NAG who performed the measurements. SPSS 15 and MS Excel 7.0 software was used for the calculation of intra-class correlation coefficients (ICC) and evaluation of 95% limits of agreement according to the procedure as described by Bland & Altman [39]. We used a two-way mixed model to calculate the ICC. The ICC is the portion of the patient variance in relation to the total. An ICC close to 1 means that two measurement methods have a high level of agreement. An ICC close to 0 shows that there is a lot of variation between two methods and a low level of agreement equivalent to be expected by chance. In accordance with Landis and Koch, the following ICC interpretation scale was used: poor to fair (below 0.4), moderate (0.41–0.60), excellent (0.61–0.80), and almost perfect (0.81–1) [40]. With Bland & Altman plots we calculated the mean difference and 95% confidence interval between two measurement methods [39].

## Results

**Figure 2:**
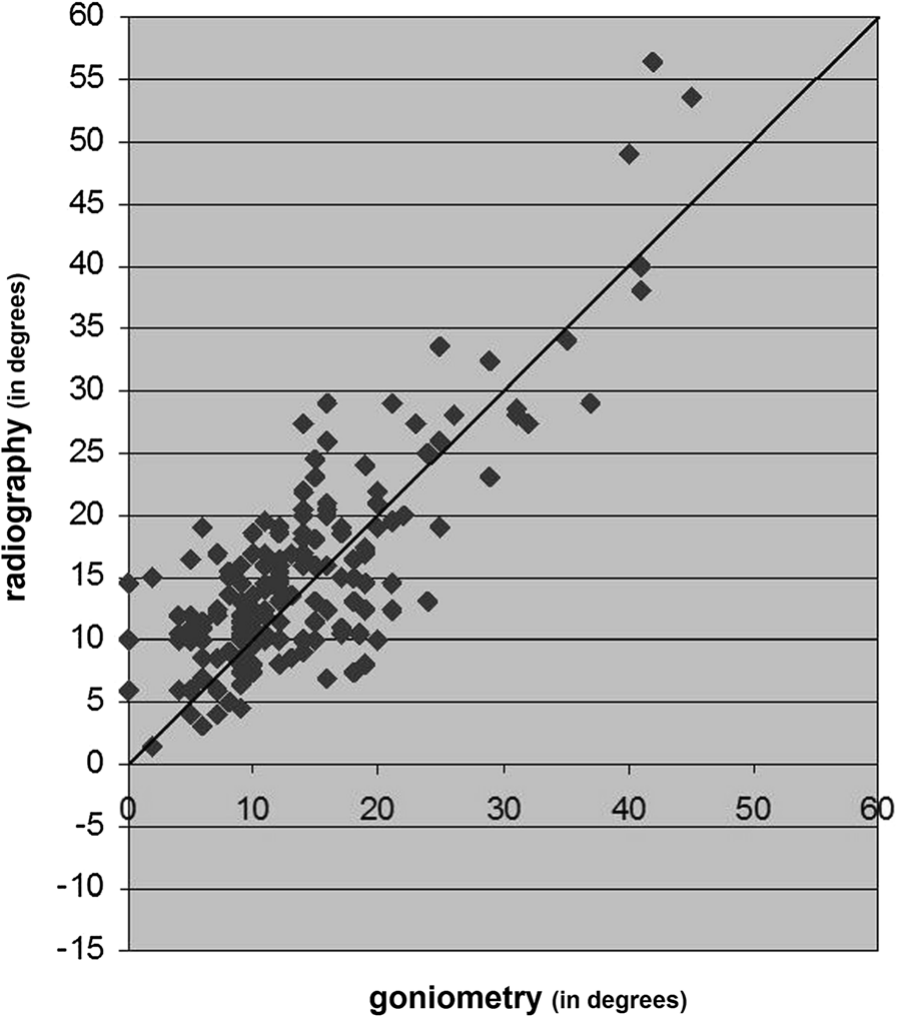
The results of radiographic measurement of HVA compared with the results of clinical goniometry of HVA.

**Figure 3:**
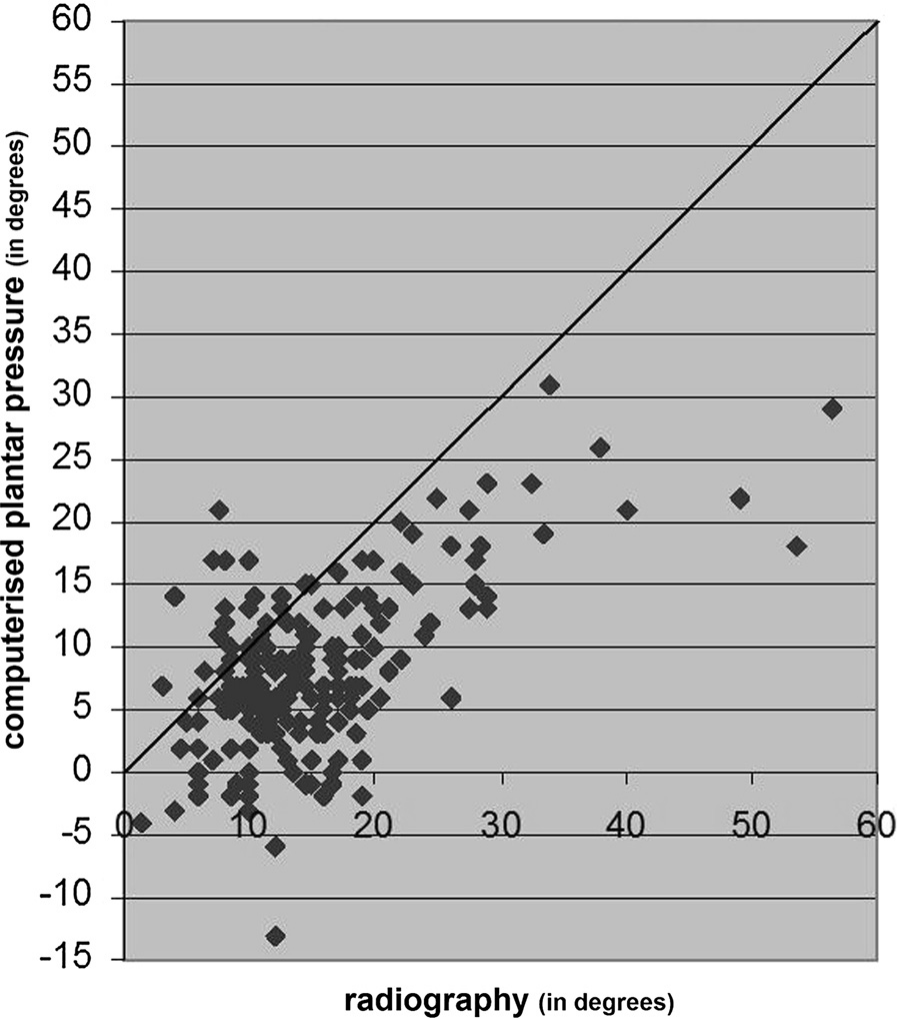
The results of computerised plantar pressure measurement of HVA compared with the results of radiographic measurement of HVA.

**Figure 4:**
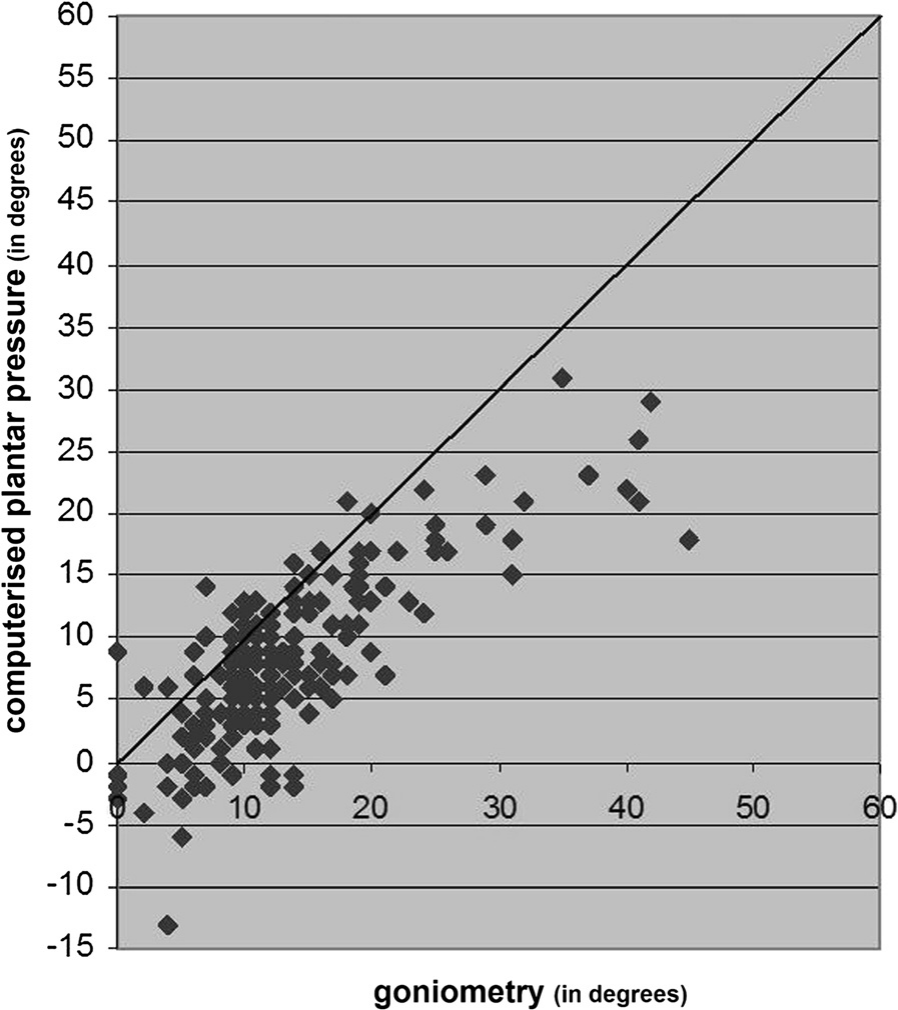
The results of computerised plantar pressure measurement of HVA compared with the results of clinical goniometry of HVA.

In this study we used data of the right foot of 186 participants with diabetes with a mean age of 58 years and mean diabetes duration of 15 years. Figures 2, 3 and 4 present the results of the three measurements in comparison to each other.

**Figure 5:**
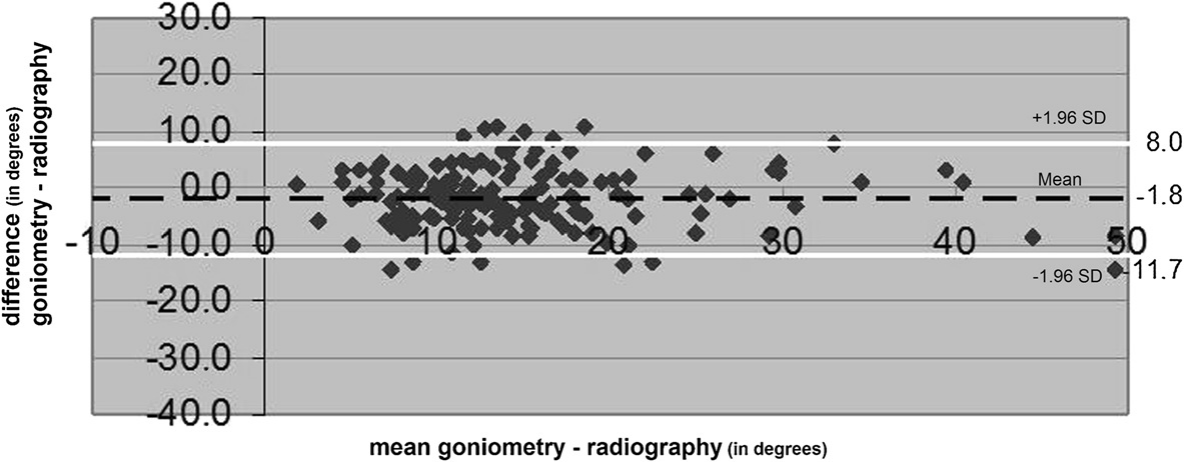
**The range of the difference between the results of clinical goniometry of HVA and radiographic measurement of HVA.** (Degrees). The mean difference (black discontinuous line) and the prediction interval (white lines) are presented.

**Figure 6:**
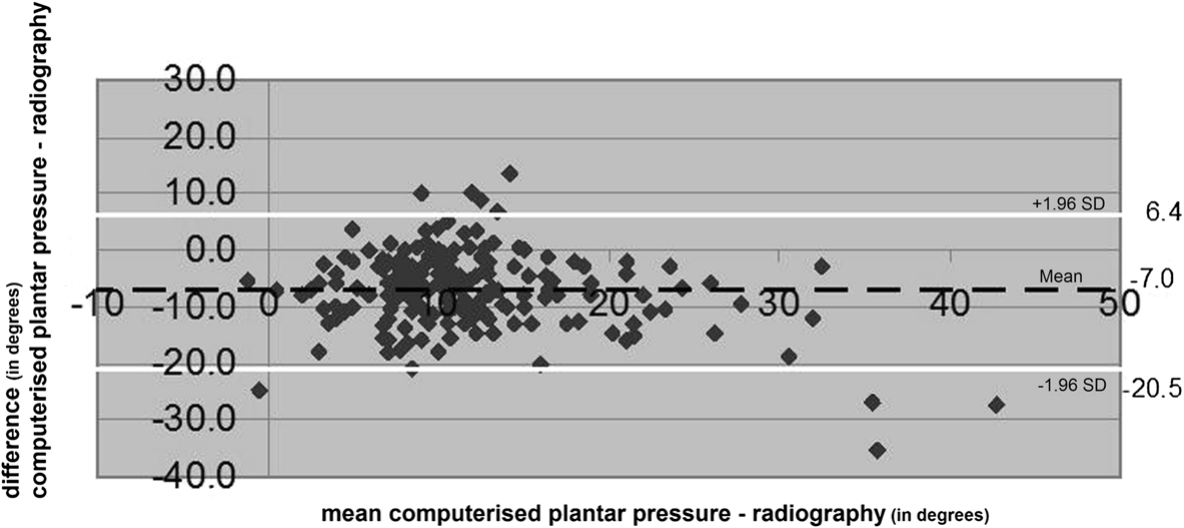
**The range of the difference between the results of computerised plantar pressure measurement of HVA and radiographic measurement of HVA.** (Degrees). The mean difference (black discontinuous line) and the prediction interval (white lines) are presented.

**Figure 7:**
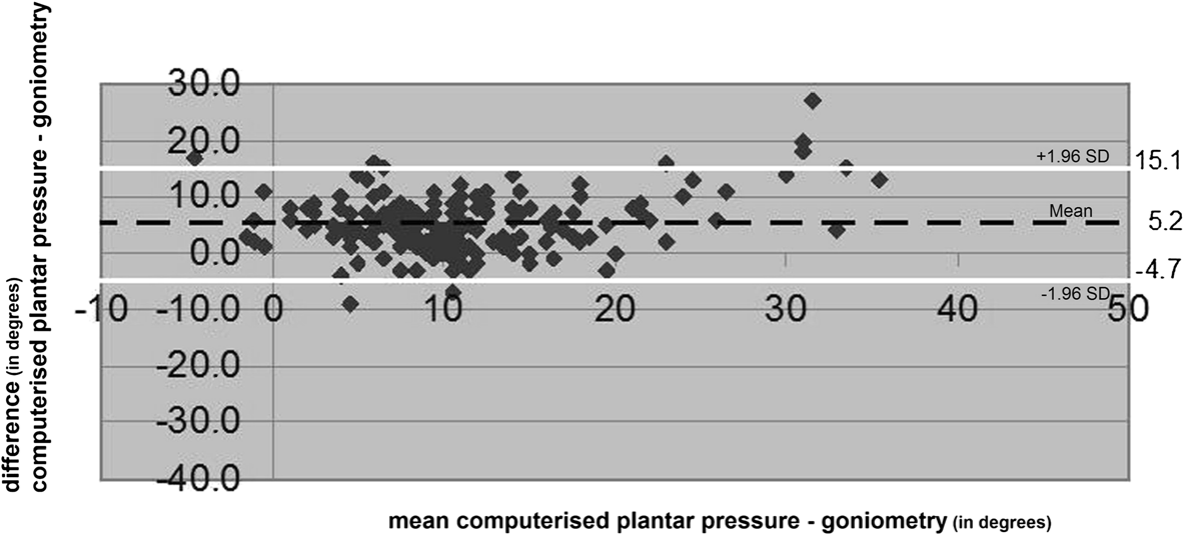
**The range of the difference between the results of clinical goniometry of HVA and computerised plantar pressure measurement of HVA.** (Degrees). The mean difference (black discontinuous line) and the prediction interval (white lines) are presented.

The mean hallux valgus deviation was 12.34 degrees (SD 7.65 degrees). The mean HVA assessed with clinical goniometry was 13.46 degrees (SD 8.05; minimum 0 degrees; maximum 45 degrees). The mean HVA assessed with radiographic measurements was 15.30 degrees (SD 8.26; minimum 1.5 degrees; maximum 56 degrees). The mean HVA assessed with computerised plantar pressure measurement was 8.26 degrees (SD 6.65; minimum -13 degrees; maximum 31 degrees) (Table 1). Figures 5, 6 and 7 represent Bland & Altman plots where the difference between two measurement methods is plotted against the mean.

**Table 1:** Mean hallux valgus angle (HVA), standard deviation (SD) and range of all three measurement methods

**Measurement method**	**Mean HVA**	**SD**	**Minimum**	**Maximum**
Clinical goniometry	13.46	8.05	0.0	45.0
Radiographic measurement	15.30	8.26	1.5	56.0
Computerised plantar pressure measurement	8.26	6.65	−13.0	31.0
Mean of all three methods	12.34	7.65	−13.0	56.0

### Radiographic measurement vs. clinical goniometry

Overall, the radiographic measurements resulted in greater HVA compared to clinical goniometry (mean difference, -1.8 degrees; 95% confidence interval -1.1 degrees to -2.6 degrees) (Table 2). The range of difference of these two measurements had a prediction interval with a maximum of 8.0 degrees and a minimum of -11.7 degrees; range 19.7 degrees (Figure 5). The pertaining method agreement resulted in an ICC of 0.81 (95% confidence interval 0.76 to 0.86; *p*<0.001).

**Table 2:** Comparison between measurement methods

	**Mean difference (95% CI)**	**Mean range of difference (range)**	**ICC (95% CI)**
Radiographic measurement vs. Clinical goniometry	−1.8 (−1.1 to 2.6)	19.7 (−11.7 to 8.0)	0.81 (0.76 to 0.86)
Radiographic measurement vs. Computerised plantar pressure	−7.0 (−6.1 to −8.0)	26.9 (−20.5 to 6.4)	0.59 (0.49 to 0.68)
Clinical goniometry vs. Computerised plantar pressure	5.2 (4.5 to 5.9)	19.8 (−4.7 to 15.1)	0.77 (0.70 to 0.82)

### Radiographic measurement vs. computerised plantar pressure measurement

The radiographic measurements of HVA resulted in greater values overall, compared to computerised plantar pressure measurements (mean difference -7.0 degrees; 95% confidence interval -6.1 degrees to -8.0 degrees) (Table 2). The range of the difference of radiographic measurement and computerised plantar pressure measurement showed a prediction interval with a maximum of 6.4 degrees and a minimum of -20.5 degrees; range 26.9 degrees (Figure 6) and resulted in an ICC of 0.59 (95% confidence interval 0.49 to 0.68; *p<0.001)*.

### Clinical goniometry vs. computerised plantar pressure measurement

The mean difference of the HVA between clinical goniometry and computerised plantar pressure measurements was 5.2 degrees (95% confidence interval 4.5 degrees to 5.9 degrees) (Table 2). Clinical goniometry measured an overall average of 5.2 degrees increase compared to computerised plantar pressure measurements. The range of the difference of these two measurements had a prediction interval with a maximum of 15.1 degrees and a minimum of -4.7 degrees; range 19.8 degrees (Figure 7) and ICC 0.77 (95% confidence interval 0.70 to 0.82; *p<0.001)*.

## Discussion

In this study, the agreement of hallux valgus deviation was assessed against the gold standard and to two alternative measurements as well as between each other. The results of these comparisons show a mean difference that ranged from 1.8 to 7.0 degrees and the prediction interval of all the comparisons is at least 19.7 degrees. These findings are not according to the study results of Sanders et al. [8]. In their study a statistically significant correlation between measurements of HVA with weight-bearing radiographs and with static ink footprints in eleven participants was found. This discrepancy is possibly caused by the difference in measurements. In this study the participants were measured by computerised plantar pressure during gait. In the study of Sanders et al. static footprints were used [8]. The large difference in sample size and the different statistical method used by Sanders et al. could also contribute to the discrepancy between the findings in these two studies.

Presumably, the low level of agreement is a consequence of the reproducibility of the three measurement methods. The reliability of radiography depends on the method used [41]. Piqué-Vidal et al. [42-44] studied the agreement of a manual and a digital measurement method with an outcome of an ICC of 0.89 to measure HVA. Farber et al. [45] studied these methods with an outcome of an inter-observer agreement of 66% for the manual method and 81% for the digital method and an intra-observer agreement of 72% for the manual method versus 80% for the digital method. Schneider et al. [13,29] studied the inter and intra-observer coefficient of repeatability of radiographic measurement of HVA, with and without a protocol described by Mitchell et al. [28]. The mean intra-observer coefficient of repeatability for the HVA improved from 5.9 degrees to 4.2 degrees using this protocol [13,29]. The inter-observer coefficient of repeatability improved from 6.5 degrees to 5.0 degrees with the protocol described by Mitchell et al. [13,29].

In this study, the angles between longitudinal bone axes with radiographic measurements of HVA and the angles between lines outside the contours of the soft-tissue with clinical goniometry and plantar pressure measurement were used. In the literature, the reproducibility of clinical goniometry and computerised plantar pressure measurement for HVA is less frequently evaluated compared to the reproducibility of radiographic measurements. Clinical goniometry of hallux valgus, through the positioning of a goniometer on the medial contour of the foot or on a foot print, is appreciated as less accurate compared to measurement through longitudinal bone axes with radiography. The latter because the measurement could be troubled by irregularity of soft-tissue contours during loading of the foot or as a result of the presence of a bunion with soft-tissue swelling. The computerised plantar pressure measurement for HVA could also be influenced when the HVA degree was that big that the hallux had overridden the second toe. Furthermore, in this study we used a dynamic plantar pressure measurement. Dynamic computerised plantar pressure measurement shows systematically smaller HVAs compared to other methods. These smaller angles are possibly caused by a different alignment of the hallux during gait in comparison with static weight bearing. The primary value of a platform-based pressure distribution analysis is to document dynamic barefoot function (e.g. excessive pronation) and aberrant pressure distribution during gait. Measuring foot angles is not the primary application of a platform-based pressure analysis. The general reliability of footprint angles, not specific HVA, is low (0.33 to 0.78) compared to the reliability of plantar pressure (0.75 to 0.90) [30,32,46-48].

The limited agreement in results between the studied HVA measuring methods is relevant in the clinical decision making process as well as research. In the literature, a HVA of 0 to 15 degrees is considered to be normal [7,11,49]. According to Kelikian et al. and Vanore et al. [11,49], the HVA could be differentiated into three stages: a mild hallux valgus deviation, between 15 and 25 degrees; a moderate deviation, between 25 and 35 degrees; a severe deformity, more than 35 degrees. This classification is based on radiographic measurements. Alternative techniques, like computerised plantar pressure measurements and clinical goniometry for HVA, could not be used to classify hallux valgus deviation according to Kelikian et al. or Vanore et al. [11,49]. The poor agreement between these measurement methods and radiographic measurements limit their usefulness for clinical practice. Tang et al. [50] studied a conservative therapy in hallux valgus and concluded that pain reduced and walking ability improved significantly when the HVA was reduced by a mean of 6 to 7 degrees. These data suggest that a difference of approximately 5 degrees could have a clinically relevant influence on therapy and symptoms. Thus concluding a measurement which deviates that is will influence therapy, as seen in our study, is not acceptable. For podiatrists who do not have radiographic facilities, it is a better alternative to use validated categorical rating scales compared to the use of clinical goniometry or computerised plantar pressure measurements [7,19,20,22].

A limitation of this study is that only one investigator performed the clinical measurements. Data generated by this study were not appropriate for assessing the intra- and inter-observer variation. The limited reproducibility of all three measurement methods as described in the literature limits the level of agreement between these methods [40,42,43,51-53]. In order to increase the reproducibility of clinical goniometry for HVA we suggest the development of a reproducible and valid standardised procedure using this measurement. Another suggestion is to explore source variation and systematic errors in software for footprints in computerised plantar pressure measurement for HVA. In addition, a strategy to reduce the variation, e.g. through repeated measures, could be formulated. In the literature or in the user’s manual a description of limitations for the use of footprints in the computerised plantar pressure measurement for HVA was not found. However, in case of a large HVA with a hallux overriding the second toe, or with a large soft tissue swelling at the medial side of the first metatarsal head, the reproducibility is probably decreased. The scatterplot of Figure 5 indicates that underestimation in measurement through computerised plantar pressure measurements compared to radiographic measurement of HVA is greater at larger angles. Information regarding the scale dependent accuracy of measurement or so called ‘longitudinal validity’ was not found in the literature. This could form a subject for future research. Possibly, an improvement of the reproducibility of the three measurements might lead to an increase of the level of agreement between radiographic measurements of HVA and the other two measurements. Only then it might become safe for clinicians without radiographic facilities to use clinical goniometry or computerised plantar pressure measurement for HVA and use these measures in the selection and evaluation of interventions for hallux valgus.

The literature showed no arguments that the presence of diabetes in the study population have influenced the method or results. However, a possible next step could be to examine these measurement methods in a more general population.

## Conclusions

The range of the mean difference, between the three measurement methods for the hallux valgus angle is 19.7 degrees or more. This could result in unacceptable measurement errors and unreliable decision making for treatment. Therefore, computerised plantar pressure measurement or clinical goniometry, in its present form, should not be used as an alternative for radiographic measurement of HVA. As an additional indicative measurement it might be considered to use these methods for evolutionary studies, once a radiographic measurement is done.

### Consent

Written informed consent was obtained from all patients for the publication of this report and any accompanying images.

## Abbreviations

HVA: Hallux valgus angle; SD: Standard deviation; ICC: Intra-class correlation coefficients; VPT: Vibration perception threshold; BMI: Body mass index; HBa1c: Glycosylated haemoglobin; kV: Kilo voltage; mAs: Milliampere second; MTP: Metatarsophalangeal; SPSS 15: Statistical Package for the Social Sciences 15; NRS 11: Numerical rating scale 11.

## Competing interests

The authors declare that they have no competing interests.

## Authors’ contributions

NAG and GHIMW designed and wrote the research proposal. NAG collected and prepared the data. Statistical analysis was executed by DMCJ and NAG. Data interpretation was done by NAG, DMCJ and APS. Writing of the scientific paper was performed by DMCJ, APS and NAG. JH, GHIMW and LWR read and discussed the manuscript. All authors read and approved the final manuscript.
